# *Bartonella henselae* DNA detection in patients with type 1 leprosy reactions for more than six months

**DOI:** 10.1016/j.bjid.2024.103743

**Published:** 2024-04-30

**Authors:** Luciene Silva dos Santos, Lais Bomediano Souza, Isabela Maria Bernardes Goulart, Marina Rovani Drummond, Paulo Eduardo Neves Ferreira Velho

**Affiliations:** a Universidade Estadual de Campinas (UNICAMP), Faculdade de Ciências Médicas, Laboratório de Pesquisa Aplicada em Dermatologia e Infecção por Bartonella, Campinas, SP, Brazil; b Pontifícia Universidade Católica de Campinas (PUC-Campinas), Departamento de Medicina, Campinas, São Paulo, SP, Brasil; c Universidade de Uberlândia, Uberlândia, MG, Brazil; d Universidade Estadual de Campinas (UNICAMP), Faculdade de Ciências Médicas, Departamento de Medicina, Divisão de Dermatologia, Campinas, SP, Brazil

**Keywords:** Bartonella, Coinfection, Leprosy

## Abstract

Leprosy reactions are among the main causes of physical disability resulting from an infectious disease and can culminate in irreversible physical disabilities, therefore they should be considered a clinical emergency, as well as the elucidation of its cause. Co-infections are considered one of the main triggering causes of leprosy reactions, aggravating and maintaining these reactions for longer in these patients. After reporting a high rate of Bartonella henselae infection in patients with chronic type 2 leprosy reaction, 19/47 (40.4 %) compared to the control group, 9/50 (18.0 %), *p* = 0.0149, we conducted this study to observe the rate of infection by Bartonella sp. in a group of patients with chronic type 1 leprosy reactions. Blood samples from 14 patients with chronic type 1 leprosy reactions were analyzed by molecular and microbiological tests and compared. The results showed that, like patients with chronic type 2 leprosy reactions, this group of patients has a high proportion of *B. henselae* infection 6/14 (42.9 %), *p* = 0.88. We conclude that these bacteria can trigger chronic leprosy reactions and should be investigated in all chronic leprosy reactions patients.

*Summary Line:* Our results showed that, like patients with chronic type 2 leprosy reactions, this group of patients has the same proportion of *B. henselae* DNA detection 6/14 (42.9 %), *p* = 0.88.

Leprosy reactions are acute and commonly self-limited phenomena, presenting an inflammatory exacerbation of the host with antigens of leprosy agents, *Mycobacterium leprae* and *Mycobacterium lepromatosis.*^1^^,^^2^ These reactions can be classified as Type 1 (T1R) ‒ often called reverse reactions ‒ and Type 2 (T2R), often clinically expressed as Erythema Nodosum Leprosum (ENL). Typically, these reactions manifest between the first six and 12 months of treatment but can occur at any time of the disease ‒ before diagnosis, during, and years after treatment.^2^^,^^3^ Clinically, T1R emerge with new skin lesions and/or worsening of pre-existing ones, worsening of the sensory and motor neurological condition, intensification of pain in peripheral nerves, and/or edema of the feet and hands. They are not accompanied by erythematous nodules or systemic symptoms. Eventually, TR1 and TR2 can coexist.^2^ Although T2R last approximately two weeks, T1R tend to endure further, lasting from weeks to months.^3^

Immunopathogenicity is related to the duration of the reactions. Although not fully clarified, it is known that in T1R occur a predominance of the pattern of cellular response of Th1 lymphocytes, releasing interleukin-2 and beta, Tumor Necrosis Factor (TNF), and Interferon Gamma (IFN**-γ**).^4^ Patients harboring cellular immunity against *M. leprae* may manifest Type 1 Reaction (T1R), particularly those categorized as borderline leprosy. These aspects are often responsible for severe and disabling neurological sequelae.^5^

Chronic leprosy reactions are considered uncommon events. The medical literature defines chronic T2R as subentrant reactions lasting longer than six months.^6^ There is no consensual classification of chronicity for T1R in the literature, but enduring forms are well known and can cause severe neurological sequelae, being necessary the use of corticosteroid treatment for a long period, often without a complete control of reactions.^7^^,^^8^

T1R, as well as T2R, are triggered by interferences in the host's immune system. Vaccines, pregnancy, puerperium, chemotherapy, infestations, and concomitant infections are described as triggers for leprosy reactions.^4^ Thus, infections - even if asymptomatic - should be investigated as risk factors for the development, worsening, or maintenance of T1R and T2R.^9^ In a review study of the majority of cases (89 %), leprosy is diagnosed before co-infection.^10^

Recent studies have associated *Bartonella henselae* infection with chronic T2R. Santos et al. described the case of a patient with multibacillary leprosy with subentrant T2R for years in which the diagnosis and treatment of *B. henselae* infection changed the occurrence of leprosy and its reactions.^9^ This index case led to the comparison of the prevalence of *Bartonella* spp. in patients with chronic T2R and volunteers without clinical symptoms. Detection of *B. henselae* DNA was possible in 19 of the 47 patients (40.4 %) and a statistical difference was observed when compared to the control group (*p* = 0.0149).^11^

This pilot study aimed to evaluate the occurrence of *Bartonella* spp. detection in patients with T1R for more than six months and to compare the outcome with the study addressing T2R.^11^

The project was submitted to the Research Ethics Committee of the Federal University of Uberlandia/UFU (CAAE: 44670015.4.3001.5152).

Patients aged 18 years or older, non-pregnant, with non-lepromatous leprosy, and T1R clinic (exacerbation of pre-existing lesions and/or neuritis, without ENL or systemic symptoms) for more than six months were included.

The same methodology described in the study with patients with chronic T2R was used.^11^ A microbiological (with liquid and solid culture) and molecular (with conventional genus-specific PCR for the Internal Transcribed Spacer [ITS] region, nested *B. henselae* species-specific PCR for the *ftsZ* gene and qualitative genus-specific real-time PCR for the *gltA* gene, both last species-specific for *B. henselae*) assays were conducted based on collected blood samples.

In total, 14 patients with T1R for more than six months monitored at CREDESH (National Reference Center for Sanitary Dermatology and Leprosy, Hospital de Clínicas, Federal University of Uberlandia ‒ MG, Brazil) were included in the investigation.

*B. henselae* DNA was detected in six patients (42.9 %) of the study group. All patients with DNA detection in the reactions presented at least one species-specific reaction for *B. henselae*. It was possible to obtain isolates from two patients. Using the Sanger method, we were able to sequence four amplified nucleotides, all presented a similarity of 99 % to 100 % for *B. henselae*. The access number corresponding to the GenBank® found for the *ftsZ* region was HG965802.1.

We found that patients with chronic type 1 leprosy reactions, as well as patients with chronic type 2 leprosy reactions, have a high rate of *B. henselae* DNA detection in patients´ blood samples (Table 1).

**Table 1:** Comparison of *Bartonella henselae* DNA detection among patients with type 1 leprosy reactions and patients with type 2 leprosy reactions for more than six months.

**Chronic leprosy reactions**			
Group	Type 1	Type 2	*p*-value^a^
Detection of *Bartonella henselae* DNA (%)	6 (42.9)	19 (40.4)	0.88
Total	14	47	

a Chi-Square.

Leprosy reactions are often difficult to control. Finding the triggering or maintaining factor of the reactions is very important to minimize the morbidity of patients and prevent sequelae, so common and severe, in patients with reactional neuritis of both T1R and T2R.^4^^,^^5^ Patients with T1R are difficult to treat and only 70 % of patients respond to corticosteroid treatment.^7^ Similar to the T2R classification,^6^ in this study we consider as chronic reaction patients those with six months of T1R without the possibility of removing immunomodulatory drugs for the control of reactions or those who have their reactions uncontrolled even with immunosuppressive treatment.

Similar to leprosy, bartonellosis is supposed to be more prevalent in vulnerable populations.^2^^,^^5^

The potential for *B. henselae* to be the trigger for chronic leprosy reactions has already been shown in patients with chronic T2R.^9^^,^^11^ This study shows that detection of this bacterium DNA was also possible in patients with T1R for more than six months.

*B. henselae* has universal distribution. Cats and dogs are reservoir for these bacteria that are, therefore, close to humans, in whom they can cause asymptomatic infection, as observed in donors. Furthermore, *B. henselae* is the main species of *Bartonella* to cause diseases in humans and, as demonstrated by Santos et al., is also related to leprosy reactions.^9^

Based on this pilot study, it is possible to conclude that patients with T1R for more than six months have detection of *B. henselae* DNA in the same proportion as patients with chronic T2R.

## Conclusions

Leprosy reactions are the main cause of infectious physical disability. Coinfections are one of the main triggers of leprosy reactions, as well as their maintenance and increased possibility of physical damage. Our studies showed that *Bartonella henselae* DNA was detected in blood samples from patients with chronic type 1 leprosy reactions, as well as in patients with chronic type 2 leprosy reactions (40 %). Therefore, *Bartonella henselae* infection should be investigated in all patients with chronic leprosy reactions.

## Funding

Doctoral scholarship by The Brazilian National Council for Scientific and Technological Development (CNPq) 170501/2018-3 (Santos, LS); Postdoctoral Scholarship by The São Paulo Research Foundation (FAPESP) 2018/12565-6 (Drummond, MR); Productivity Grant by The Brazilian National Council for Scientific and Technological Development (CNPq) 306970/2018-0 (Velho, PENF); Research Grant by the São Paulo Foundation against Leprosy 193/2018; Support Fund for Dermatology of São Paulo (FUNADERSP) 85-2019; Fund for Support to Teaching, Research and Outreach Activities (FAEPEX) 3184/18.

## Conflicts of interest

The authors declare no conflicts of interest.
